# Experimental Bioactive Glass-Containing Composites and Commercial Restorative Materials: Anti-Demineralizing Protection of Dentin

**DOI:** 10.3390/biomedicines9111616

**Published:** 2021-11-04

**Authors:** Matej Par, Andrea Gubler, Thomas Attin, Zrinka Tarle, Andro Tarle, Tobias T. Tauböck

**Affiliations:** 1 Department of Endodontics and Restorative Dentistry, School of Dental Medicine, University of Zagreb, Gunduliceva 5, 10000 Zagreb, Croatia; tarle@sfzg.hr; 2 Department of Conservative and Preventive Dentistry, Center for Dental Medicine, University of Zurich, Plattenstrasse 11, 8032 Zurich, Switzerland; andrea.gubler@zzm.uzh.ch (A.G.); thomas.attin@zzm.uzh.ch (T.A.); tobias.tauboeck@zzm.uzh.ch (T.T.T.); 3 Community Health Center Zagreb—Center, Runjaninova 4, 10000 Zagreb, Croatia; andro.tarle@gmail.com

**Keywords:** experimental resin composites, remineralizing dental materials, bioactive glass, glass ionomer, alkasite, giomer

## Abstract

The purpose of this in vitro study was to investigate whether different types of experimental and commercial restorative dental materials can protect dentin against acid-induced softening. Experimental composites were prepared with a photocurable mixture of methacrylates and two types of bioactive glass (45S5 and a customized low-Na F-containing formulation). Human dentin samples were prepared from mid-coronal tooth slices and immersed in lactic acid solution (pH = 4.0) at 5 mm from set specimens of restorative material. After 4, 8, 12, 16, 20, 24, 28, and 32 days, surface microhardness of dentin samples and pH of the immersion solution were measured, followed by replenishing of the immersion medium. Microstructural analysis was performed using scanning electron microscopy. The protective effect of restorative materials was determined as dentin microhardness remaining statistically similar to initial values for a certain number of acid additions. Scanning electron microscopy showed a gradual widening of dentinal tubules and proved less discriminatory than microhardness measurements. To produce a protective effect on dentin, 20 wt% of low-Na F-containing bioactive glass was needed, whereas 10 wt% of bioactive glass 45S5 was sufficient to protect dentin against acid-induced demineralization. The anti-demineralizing protective effect of experimental and commercial restoratives on dentin was of shorter duration than measured for enamel in a previous study using the same experimental approach.

## 1. Introduction

Composite materials based on methacrylate resins and glass fillers are used for a wide variety of dental applications [1]. Besides their main application for restoring damaged dental hard tissues, this versatile material class is used in prosthodontics for luting of indirect restorations, in orthodontics for bonding of brackets, and in pedodontics for minimally invasive and preventive treatments.

The main shortcoming of all dental resin composites originates from their volumetric shrinkage during polymerization [2]. After the material is applied into the tooth cavity and its setting is triggered using blue light, the polymerization reaction leads to the shortening of intermolecular distances, consequently reducing the macroscopic material volume and ultimately resulting in localized discontinuities between the restoration and tooth cavity margin [3]. These marginal flaws are readily populated by cariogenic bacteria, facilitating their growth by providing protection from regular tooth cleansing. The accumulated bacteria create an acidic environment conducive for the formation of recurrent (secondary) caries at restoration margins [4]. This sequence of adverse events is regarded as the main mechanism for the failure of contemporary composite restorations [5].

To reduce the susceptibility of composite restorations to secondary caries, various mechanisms for hindering the events leading to its development are being investigated. The possible approaches range from designing composites characterized by reduced shrinkage stress [6] or being capable of antibacterial action [7], acid neutralization [8], remineralization of tooth tissues [9], and marginal gap-sealing [10]. Although clinical evidence of their effectiveness is lacking, most of these approaches show promising effects in vitro [11]. Among various compounds that are being investigated as potential additives to achieve the anti-caries activity of dental resin composites, bioactive glasses (BGs) appear especially promising due to their capability to simultaneously release remineralizing ions (Ca^2+^, PO_4_^3−^, F^−^), neutralize acids, and precipitate calcium phosphates on their surface [12,13,14,15,16,17].

A series of previous studies investigated experimental composites functionalized with conventional BG 45S5 and a customized low-Na F-containing BG [8,18,19,20,21]. These composites demonstrated a protective anti-demineralizing effect on human enamel blocks by maintaining their microhardness when immersed in repeatedly replenished lactic acid solution [19]. In addition to the experimental BG-containing composites, three commercial restorative materials (glass ionomer, giomer, and alkasite) also demonstrated an anti-demineralizing effect on enamel [19]. The protective effect was identified for enamel blocks that were placed 5 mm away from experimental composite samples, which represents an improvement in commonly investigated and reported protective effects on tooth tissues that are immediately adjacent to restorative material specimens [22,23,24,25]. To investigate whether such remotely acting protection against demineralization can also be achieved for dentin, the present study employed the same experimental protocol as the previous study [19], with the only difference of dentin specimens being used instead of enamel. The aims of the present study were to investigate if the experimental BG-containing composites and three commercial restorative materials can protect dentin against acid-induced softening and compare the protective effect to that reported previously for enamel.

The null hypotheses assumed no differences in dentin microhardness and pH of the immersion medium: (I) among different time points representing the cycles of repeated acid additions; and (II) among the tested materials.

## 2. Materials and methods

### 2.1. Experimental Resin Composites

The experimental BG-containing composites were based on a photocurable 60:40 wt% bisphenol-A-glycidyldimethacrylate (Bis-GMA, Merck, Darmstadt, Germany) and triethylene glycol dimethacrylate (TEGDMA, Merck, Darmstadt, Germany) resin system. The photoinitiator system consisted of camphorquinone (0.2 wt%; Merck) and ethyl-4-(dimethylamino) benzoate (0.8 wt%; Merck, Darmstadt, Germany). All components of the resin system were blended for 48 h using a magnetic stirrer.

Experimental composites were prepared by mixing the photocurable resin with the fillers listed in Table 1. BG 45S5 and silanized reinforcing fillers (inert barium glass and silica) were commercially available, while the experimental low-Na F-containing BG was prepared on-demand by Schott (Mainz, Germany) via a melt-quench route. This BG type was designed to have theoretical network connectivity similar to that of BG 45S5 (2.1) and comparable particle size distribution as the conventional BG 45S5 used in this study.

Experimental composites contained a total filler ratio of 70 wt%, which was composed of silanized reinforcing fillers (barium glass and silica) and one of two types of unsilanized BG. A fraction of reinforcing fillers (0, 10, or 20 wt%) was replaced by BG fillers, as presented in Table 2. The mixing of fillers and the resin system was performed in dark containers using a dual asymmetric centrifugal mixing system (Speed Mixer TM DAC 150 FVZ, Hauschild & Co. KG, Hamm, Germany) at 2000 rpm for 5 min. The mixed composite pastes were de-aerated in a vacuum for 48 h.

As reference materials, three contemporary restorative materials with remineralizing and acid-neutralizing capabilities were included: a reinforced glass ionomer restorative (ChemFil Rock, Dentsply Sirona, Konstanz, Germany; shade: A2, LOT: 1903000819), a giomer (Beautifil II, Shofu, Kyoto, Japan; shade: A2, LOT: 041923), and a resin-based “alkasite” material (Cention, Ivoclar Vivadent, Schaan, Liechtenstein; shade: universal, LOT: XL7102).

### 2.2. Dentin Samples

Intact human third molars (*n* = 26) were collected as by-products of regular dental treatment and irreversibly anonymized. The teeth were stored at 8 °C in 0.1% thymol solution and used within 6 months of extraction. Written informed consents were obtained from all patients who agreed to the use of their teeth in research. Hence, the present study complied with the use of anonymized biological material, and authorization from the local ethics committee was not required (Federal Act on Research Involving Human Beings (Human Research Act; article 2, paragraph 2)).

**Figure 1:**
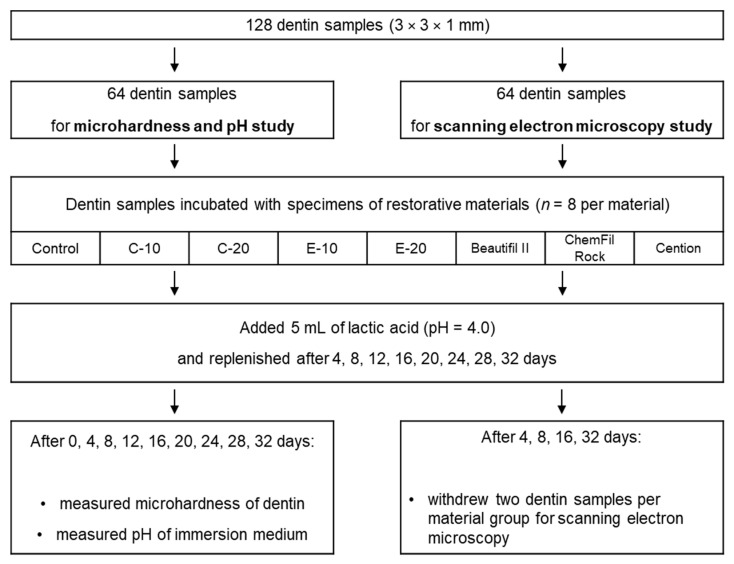
Flowchart of experimental design.

Dentin samples (3 × 3 × 1 mm; 5 samples per tooth on average) were prepared from mid-coronal tooth slices using a low-speed precision cutting machine (IsoMet, Buehler, Lake Bluff, IL, USA). The occlusal sides of dentin samples were ground using P4000 silicon carbide paper (Buehler, Lake Bluff, IL, USA; 2 min at 30 rpm, median particle size = 2.5 µm). The prepared dentin samples were stored in a phosphate-buffered saline solution and used within five days of preparation. A total of 128 dentin samples were prepared, half of which were used for the microhardness (MH) and pH measurements, while the other half was used for the scanning electron microscopy (SEM) study, as shown in the flowchart in Figure 1.

### 2.3. Restorative Material Specimens

Specimens of the experimental and reference materials were prepared in discoid polyoxymethylene molds (diameter = 7 mm, thickness = 2 mm). The materials were applied into molds, covered with polyethylene terephthalate foils, and pressed with a glass plate. The glass ionomer material was left undisturbed in the mold for 15 min, while the other materials were light-cured using a LED curing unit (Bluephase PowerCure, Ivoclar Vivadent, Schaan, Liechtenstein, radiant exitance: 1340 mW/cm^2^) for 20 s. The specimens were immersed in the solutions within 15 min after preparation. Sixteen specimens were prepared for each material, of which *n* = 8 were used for the MH and pH study, and *n* = 8 were used for the SEM evaluation.

### 2.4. Immersion in Lactic Acid Solution

Dentin samples were immersed together with restorative material specimens in closed vials (Eppendorf; Hamburg, Germany) in 5 mL of lactic acid solution (pH = 4.0). Each dentin sample was placed in an individual vial and positioned at a standardized distance of 5 mm from the set restorative material specimen. The vials were placed on a horizontal laboratory shaker at a speed of 30 rpm at a temperature of 23–24 °C. After the designated time points (4, 8, 12, 16, 20, 24, 28, and 32 days), surface MH of dentin samples and pH of the immersion solution was measured. After each measurement, the immersion solution was replaced with 5 mL of fresh lactic acid solution (pH = 4.0).

### 2.5. Microhardness Measurements

MH of dentin samples was evaluated on their occlusal sides using a digital hardness tester (model no. 1600-6106; Buehler, Lake Bluff, IL, USA) equipped with a Knoop indenter. Indentations were made immediately after removing the dentin specimens from the immersion medium using a load of 100 g and a dwell time of 15 s. For each specimen and time point, three replicate indentations were made at random positions, and their mean values were considered as a statistical unit. To avoid indentation distortions due to substrate elasticity [26], the MH evaluations were performed within 30 s after indentations were made, using a resolution of 0.015 µm. Eight dentin specimens per restorative material were evaluated (*n* = 8).

### 2.6. pH Measurements

pH measurements of the immersion solution were performed using a calibrated pH electrode (780 pH Meter, Metrohm, Herisau, Switzerland). Three replicate measurements were made for each specimen and time point and their mean values were considered as a statistical unit. Eight specimens per restorative material were evaluated (*n* = 8).

### 2.7. Scanning Electron Microscopy

SEM evaluation was performed on a separate set of dentin samples (*n* = 8 per material) that were subjected to the same lactic acid immersion protocol as the set of specimens used for the MH and pH study. At each of the following time points: 4, 8, 16, and 32 days, 2 dentin samples per restorative material were taken out of the immersion solution, rinsed with distilled water, dried, and sputter-coated with gold (5 nm). The surfaces of dentin samples were observed using a scanning electron microscope (SEM; GeminiSEM 450, Zeiss, Oberkochen, Germany) at 10 kV and 10,000× magnification.

### 2.8. Statistical Analysis

The data were assessed for the assumption of normality of distribution using Shapiro–Wilk’s test and the inspection of normal Q-Q plots. Homogeneity of variances was verified using Levene’s test. At the level of each restorative material, MH and pH values were compared among time points using repeated-measures ANOVA with Bonferroni post-hoc adjustment. An overall level of significance of α = 0.05 was used. Statistical analysis was performed using SPSS (version 25, IBM, Armonk, NY, USA).

## 3. Results

Dentin MH values measured after successive acid addition cycles are presented in Table 3. As MH values significantly decreased over time in acid immersion, the protective effect of restorative materials was determined as MH remaining statistically similar to initial values for a certain number of acid additions. For the control composite, as well as for E-10, Beautifil II, and ChemFil, dentin MH was significantly different from the baseline values already at the first measurement point (4 days). For the other materials, dentin MH was maintained over different numbers of acid additions, as follows: C-10 and E-20 (up to 4 days), Cention (up to 8 days), and C-20 (up to 12 days).

**Figure 2:**
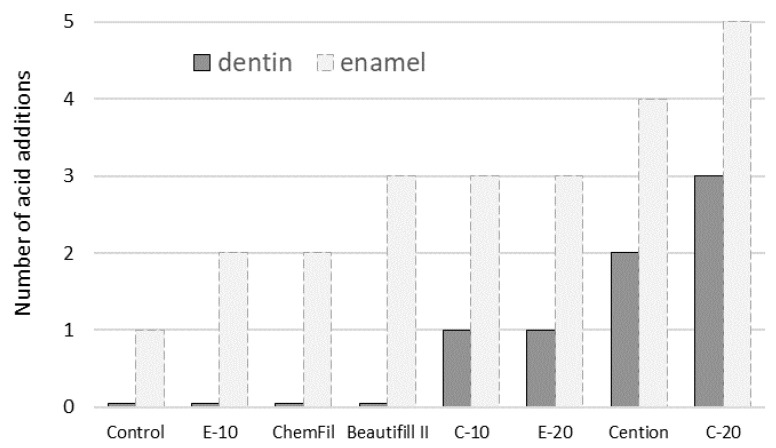
Number of acid addition cycles for which dentin surface MH remained statistically similar to initial values. Light-grey bars with dashed borders show the corresponding values measured in a previous study [19] for enamel blocks under the same experimental conditions.

The number of acid additions over which dentin MH remained unchanged (i.e., statistically similar to baseline values measured before acid immersion) is summarized in Figure 2. To allow a direct comparison with the data reported in a previous study on enamel blocks [19], an additional data series for the same parameter from that study is shown in Figure 2.

**Figure 3:**
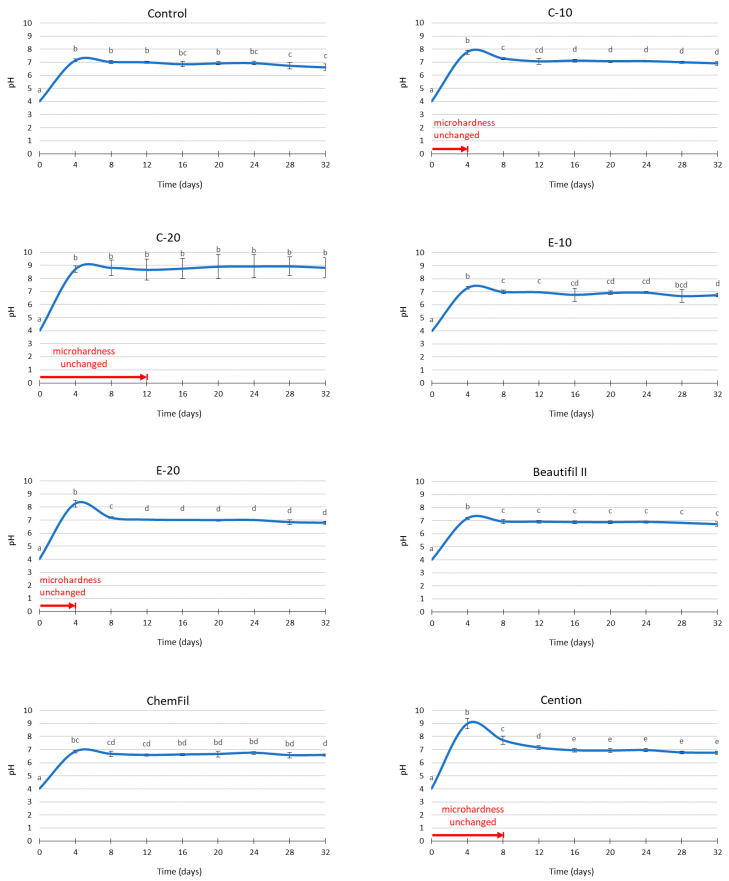
pH changes (mean values ± standard deviations) of the immersion medium and time periods for which dentin microhardness remained unchanged compared to initial values. Same letters denote statistically similar pH values within a material.

The pH values of the immersion medium are presented in Figure 3. In all experimental groups, the initial pH of the solution (before immersion of dentin samples and restorative material specimens) was 4.0. Material-dependent increases in pH values were identified in all experimental groups. By the end of the observation period of 32 days, all materials reached stable values of pH = 6–7, except C-20 which plateaued at pH = 9. At the first time point (4 days), transient peaks in pH values were observed for C-10 (pH = 8), E-10 (pH = 7.5), E-20 (pH = 8.5), Beautifil II (pH = 7.5), and Cention (pH = 9). The pH values for these materials were leveled at subsequent time points, reaching the aforementioned plateau values.

**Figure 4:**
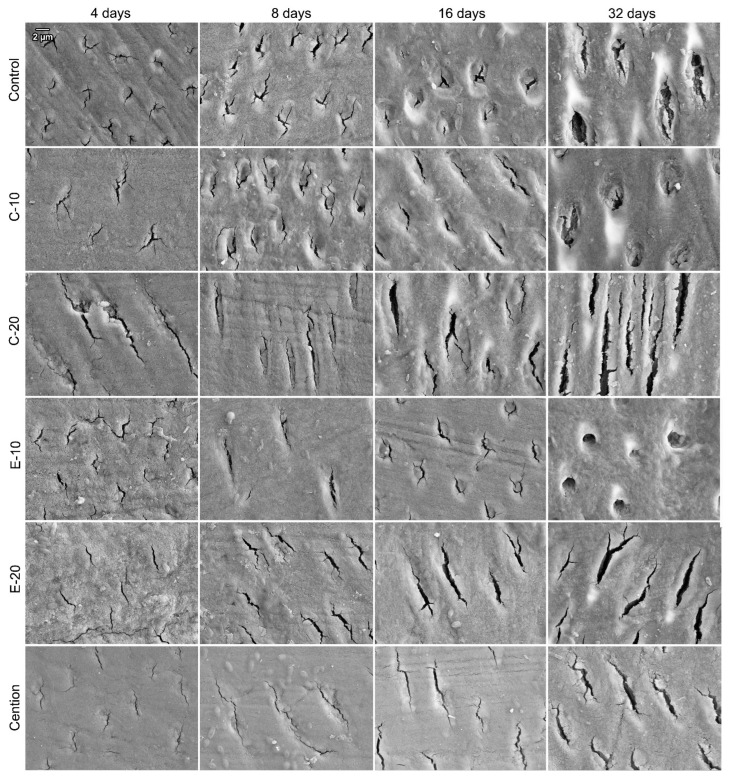
Representative scanning electron microscopy images of dentin surfaces immersed with experimental composites and one of the commercial reference materials (Cention). Due to variations in the direction of dentinal tubules relative to the plane of sectioning, the cross-sections of dentinal tubules range from circular to oblique.

Representative SEM images of surfaces of dentin samples immersed with control, C-10, C-20, E-10, E-20, and Cention are shown in Figure 4. The main morphological change observable in all experimental groups was the progressive widening of dentinal tubules over time.

## 4. Discussion

As a sequel to a previous study on the anti-demineralizing effects of restorative dental materials on enamel [19], the present study investigated whether a protective effect can also be achieved on dentin. To ensure comparability, both studies were performed using an identical experimental protocol in which specimens of dental hard tissues (enamel in the previous study; dentin in the present study) were exposed to repeated acid attacks. Expectedly, the protection against demineralization was less successful in the case of dentin, as it is a comparably less mineralized and more permeable tissue than enamel [27]. Since the restorative material type and the number of acid additions affected the outcome variables (MH and pH), both null hypotheses were rejected.

The experimental group with the control (inert) composite maintained near-neutral pH values of the immersion medium through all time points, implying that the dissolution of dentin over each of the four-day acid exposure cycles was sufficient to completely neutralize the lactic acid solution. This led to the overlap of the acid-neutralizing effect of restorative materials and acid neutralization caused by dentin dissolution, making C-20 the only material capable of raising and maintaining the pH above the neutral range. For the materials with a transient alkalization (C-10 and E-20), the period of pH increase corresponded to the period over which dentin MH remained unchanged (four days), indicating a contribution of their acid-neutralizing effect [8] on the protective effect on dentin. C-20, however, kept the dentin MH unimpaired for only 12 days, despite its continuous capability to raise the pH to 8–9 over the whole observational period. The finding that dentin MH decreased regardless of alkaline pH was explained in the previous study on enamel blocks as being the result of demineralization occurring in between the successive 4-day measurements, i.e., during the time between the fresh 5 mL of the lactic acid solution have been added and the end of each 4-day cycle when the alkaline pH was reached [19].

The previous study on enamel [19] showed that an anti-demineralizing protective effect can be attained even without alkalization of the immersion medium; this finding was attributed to the ions released from the restorative materials, which reduced the solubility of enamel in an acidic medium [21,28]. However, in the present study, no protective effect occurring independently on alkalization was observed. This was likely due to the comparatively higher sensitivity of dentin to demineralization due to its lower mineral content and higher permeability. The higher sensitivity of dentin to demineralization also explains the differences in protective capabilities between enamel and dentin shown in Figure 2, which indicates similar material rankings within a substrate (enamel or dentin), but a consistently poorer protective performance in dentin. The rankings of the protective effect duration in Figure 2 also show that only the four best-performing materials in the study on enamel (C-10, E-20, Cention, and C-20) were capable of demonstrating identifiable protection of dentin.

SEM images of dentin surfaces recorded after 4, 8, 16, and 32 days show a gradual widening of the lumina of dentinal tubules. This microscopic feature is consistent with gradual demineralization over the lactic acid immersion, which occurred for all experimental groups [29]. However, subtle inter-material differences identified through MH measurements were not distinguishable in SEM micrographs due to the qualitative nature of this evaluation. The SEM images revealed that not all dentin samples had dentinal tubules cut perpendicularly but rather at different angles. This originated from the natural variations in biological material and presented an unavoidable source of variability for MH measurements. Randomized allocation of the dentin samples into experimental groups helped to disperse this variability equally across all experimental groups.

A simplified model of acid attack was used for comparability with the previous study performed on enamel samples [19]. The processes of demineralization and remineralization in the dentin are comparatively more complex due to its structure characterized by the mineralized collagen network, which is more difficult to remineralize surpassing a certain amount of mineral loss [30]. Additionally, the intensity of acid attack used in the present study was exaggerated in comparison to clinically realistic acid quantities produced by oral bacterial biofilms. Nevertheless, the present study and its complementary prequel study [19] showed that the beneficial effects of acid neutralization and ion release generated by restorative materials are not limited to the immediately adjacent dental tissues, but can reach other sites within the oral cavity, e.g., caries-prone restoration margins on adjacent teeth, cavity bottoms of deep restorations with marginal leakage, and non-carious cervical/root lesions with exposed dentin and cementum.

When discussing the potential benefits of ion-releasing composites, the accompanying shortcomings of partially replacing reinforcing fillers with reactive fillers should be noted. For example, adding reactive fillers has been shown to impair mechanical properties [31] and bond strength to dentin [32]. Additionally, surface roughness can be diminished by the dissolution of reactive fillers, leading to increased bacterial accumulation [33]. These negative effects may offset the remineralizing and protective benefits of the BG-functionalized composites. As the beneficial effects and shortcomings caused by the introduction of reactive fillers are dose-dependent, further investigations of remineralizing materials should consider fine-tuning and balancing these two opposing groups of material properties.

## 5. Conclusions

The anti-demineralizing protective effect of experimental and commercial restorative materials on dentin was of shorter duration than measured for enamel in a previous study with the same experimental approach. To produce a protective effect on dentin, 20 wt% of low-Na F-containing bioactive glass was needed, whereas 10 wt% of bioactive glass 45S5 was sufficient to protect dentin against acid-induced demineralization. Unlike the previous study on enamel, which showed the protective effect for multiple commercial restorative materials (glass ionomer, giomer, and alkasite), the present study identified the anti-demineralizing effect on dentin only for the alkasite material.

## Figures and Tables

**Table 1:** Bioactive glass and reinforcing fillers used in experimental composites.

	Bioactive Glass 45S5	Experimental Fluoride-Containing Bioactive Glass	Inert Barium Glass	Silica
Particle size (d50)	3 µm	3 µm	1 µm	5–50 nm
Composition (wt%)	45.0% SiO_2_ 24.5% CaO 24.5% Na_2_O 6.0% P_2_O_5_	33.5% SiO_2_ 33.0% CaO 10.5% Na_2_O 11.0% P_2_O_5_ 12.0% CaF_2_	55.0% SiO_2_ 25.0% BaO 10.0% Al_2_O_3_ 10.0% B_2_O_3_	>99.8% SiO_2_
Silanization (wt%)	none	none	3.2	4–6
Manufacturer	Schott, Mainz, Germany	Schott, Mainz, Germany	Schott, Mainz, Germany	Evonik, Hanau, Germany
Product name/LOT	G018-144/M111473	experimental batch	GM27884/Sil13696	Aerosil R 7200/157020635

**Table 2:** Composition of experimental composites.

Material Designation	Filler Composition (wt%)			Total Filler Ratio (wt%)
	Bioactive Glass 45S5	Experimental Fluoride-Containing Bioactive Glass	Reinforcing Fillers (Inert Barium Glass: Silica = 2:1)	
**Control**	0	0	70	70
C-10	10	0	60	70
C-20	20	0	50	70
E-10	0	10	60	70
E-20	0	20	50	70

**Table 3:** Knoop microhardness of dentin samples (mean values with standard deviations in parentheses).

Time	No. of Acid Addition Cycles	Material							
		Control	C-10	C-20	E-10	E-20	Beautifil II	ChemFil	Cention
Initial	0	48.9 (1.5) a	50.2 (4.2) a	51.8 (2.7) a	53.6 (2.3) a	52.5 (2.4) a	51.6 (3.5) a	48.9 (4.4) a	53.0 (3.8) a
4 days	1	42.6 (1.9) b	45.7 (4.9) ab	48.7 (2.1) ab	46.9 (2.9) b	47.7 (4.7) ab	45.6 (3.5) b	42.2 (3.8) b	51.2 (4.5) a
8 days	2	36.7 (1.4) c	39.4 (5.5) bc	48.7 (2.3) ab	44.0 (2.6) bc	44.3 (4.3) b	40.6 (4.0) c	36.4 (3.9) c	48.1 (4.8) ab
12 days	3	33.5 (2.6) c	34.3 (5.3) c	45.6 (3.6) abc	40.8 (3.1) c	38.9 (4.3) c	36.1 (3.4) c	32.1 (2.7) c	43.3 (5.6) b
16 days	4	22.6 (2.9) d	23.7 (5.0) d	37.9 (6.4) cd	26.7 (3.5) d	27.3 (2.0) d	23.8 (2.8) d	22.0 (3.1) d	32.6 (3.4) c
20 days	5	18.4 (2.4) e	23.2 (5.1) d	41.8 (5.4) bcd	25.5 (3.6) d	24.8 (3.5) d	23.0 (2.8) d	21.4 (1.9) d	30.1 (4.6) cd
24 days	6	18.5 (3.1) e	21.2 (4.6) de	39.3 (6.2) cd	23.3 (3.1) de	24.5 (2.8) d	21.1 (1.9) d	20.0 (1.7) d	28.0 (2.8) cd
28 days	7	17.4 (2.4) e	17.0 (3.0) de	34.8 (6.6) d	20.0 (2.8) ef	22.1 (1.8) de	19.2 (2.6) de	18.1 (2.4) de	24.1 (2.5) de
32 days	8	15.1 (1.2) e	15.7 (2.8) e	34.3 (8.1) d	16.6 (2.5) f	17.0 (2.3) e	15.8 (2.1) e	14.5 (1.6) e	21.6 (2.3) e

Same letters denote statistically similar microhardness values within a material.

## Data Availability

The datasets generated during and/or analyzed during the current study are available from the corresponding author on reasonable request.
